# Supplementary material for: Quantum Calculation of the Collision-Induced Line-Shape Effects in Antiprotonic Helium and the New Accurate Ab Initio p̅He+ –He Potential Energy Surface
Source: J Chem Theory Comput. 2025 Dec 28;22(1):488–501. doi: 10.1021/acs.jctc.5c01636 (PMC12805563; doi:10.1021/acs.jctc.5c01636)
Supplement: Supplementary file 2 [file ct5c01636_si_002.pdf]

Description of Supplementary Material

"Quantum calculation of the collision-induced line-shape effects in antiprotonic helium and the new accurate ab initio $\bar{p}He^+$–He potential energy surface"

Hubert J. Jóźwiak,[1,*] Dimitar Bakalov,[2] Michał Przybytek,[3] Michail Stoilov,[2] and Piotr Wcisło[1]

[1] *Institute of Physics, Faculty of Physics, Astronomy and Informatics, Nicolaus Copernicus University in Toruń, Grudziądzka 5, 87-100 Toruń, Poland*

[2] *Institute for Nuclear Research and Nuclear Energy, Bulgarian Academy of Sciences, 1040 Sofia, Bulgaria*

[3] *University of Warsaw, Faculty of Chemistry, Pasteura 1, 02-093 Warsaw, Poland*

*Email: hubert.jozwiak@umk.pl

The Supplementary Material for this article contains the following:

(i) "**potential_energy_surface**" This directory provides *ab initio* potential energy surface for exotic helium-helium system. The potential energy surface is provided through a `FORTRAN` code ("**potcheck.f90**"), which takes as input all other files in this directory. Contents of these files and their relation to quantities introduced in the manuscript are described on the next page.

(ii) "**generalized_cross_sections**" This directory contains generalized cross-sections calculated for 50 electric dipole transitions in $\bar{p}^4He^+$ perturbed by collisions with atomic helium (^4He). The data is located in 50 *.csv files corresponding to 50 considered transitions (see Tab. 1 in the manuscript). Names of the file encode spectroscopic notation for each transition, i.e. `0-0_P31.csv` corresponds to the spectroscopic transition in which antiprotonic helium changes its state from $v = 0, j = 31$ to $v = 0, j = 30$ (see the text for details), and the `3-5_P34.csv` corresponds to the transition between $v = 3, j = 34$ and $v = 5, j = 33$ levels in $\bar{p}^4He^+$. Each csv file contains 4 columns: collision energy (`Ekin`, in cm^{-1}), inelastic contribution to the pressure broadening cross-section (`Inelastic contribution to PBXS`, in Å^2), pressure broadening cross-section (`PBXS`, in Å^2), and pressure shift cross-section (`PSXS`, in Å^2); see Eq. (17) in the manuscript. These files cover relative collision energies ranging from 10^{-3} cm^{-1} to 110 cm^{-1}.

(iii) "**line_shape_parameters**" this directory contains line-shape parameters (Eq. (16) in the manuscript): pressure broadening coefficient (γ_0) and pressure shift coefficient (δ_0) as a function of temperature, ranging from 1.5 to 15 K. The data is stored in 50 *.csv files corresponding to 50 considered transitions. The naming convention follows the one outlined in point (ii). Data is organized in 3 columns: temperature (`Temperature`, in K), pressure broadening coefficient (`Gamma0`, in cm^{-1} atm^{-1}), pressure shift coefficient (`Delta0`, in cm^{-1} atm^{-1}).

Potential energy surface. The explicit expressions of each of the three components of the interaction energy (Eq. (5) in the manuscript) – $V_S(R, r, \theta)$, $V_M(R, r, \theta)$, an $V_L(R, r, \theta)$ – involve a number of parameters. Their numerical values are given in ASCII files as electronic supplement to the present paper. Here we give the detailed description of these files needed for efficiently using them in further calculations.

The file `linpars.dat` is a list of the non-zero coefficients p_j in the expansion (Eq. (15) in the manuscript) of $V_{\rm M}(R, r, \theta)$. The file has 1040 lines; the j-th line contains the integers k_j, m_j, l_j, and the values of $p_j, j = 1, \ldots, 1040$.

The file `nonlinpars.dat` is a list of the optimized values of the 6 non-linear parameters $q_1, \ldots, q_6$, one value per line.

The optimized numerical values of the parameters in the explicit expressions of the functions $\widetilde{B}_{n,l}(r)$ in Eq. (31) are given in the files `b[n][l].dat`, e.g. `b60.dat` for the file with the parameters of $\widetilde{B}_{6,0}(r)$, etc. All these files are structured uniformly:

– each of the first two lines contains the index k and the value of a_k;
– each of the following eight lines contains the indices k and i, and the value b_{ki};
– the next (11-th) line contains the number of elements $|\mathcal{N}|$ in the set $\mathcal{N}$;
– each of the following $|\mathcal{N}|$ lines contains the values of ν and c_ν;
– the last line contains the values of the damping parameter η.

To avoid any misunderstanding, in the table below we give in explicit form the sets $\mathcal{N}$ for each of the functions $\widetilde{B}_{n,l}(r)$ of interest, together with information about the achieved fractional accuracy of the fits.

The file `control_points.dat` lists a set of 27 test grid points $(R_i, r_i, \theta_i) = 1, \ldots, 27$. This list is read by the simple program `main.f90`, which calls the `newpot` subroutine contained in the `potcheck.f90` file, to compute the values of $V_{\rm fit}(R_i, r_i, \theta_i)$ at these test points, and saves the results to `computed.dat`. These values could help verify user's codes for the evaluation of $V_{\rm fit}(R_i, r_i, \theta_i)$. For reference, we also provide the file `ref_computed.dat`, which contains the output of this test program.

To compile this example, use

```
gfortran -o potential.x main.f90 potcheck.f90
```

and run it with

```
./potential.x
```

The output can then be compared against `ref_computed.dat`.

Table S1: The set $\mathcal{N}$ of positive integer values of ν in the second term of the expression Eq. (31) for the fitting functions $\widetilde{B}_{n,l}$, and the corresponding values of the functional $\mathcal{F}[B_{n,l}(r)]$ obtained by summing up in Eq. (32) the contribution from all data points in D (last column), or from the reduced data set D' (third column).

$\widetilde{B}_{nl}$	$\mathcal{N}$	$\mathcal{F}[\widetilde{B}_{n,l}(r)]$ for fitting points	$\mathcal{F}[\widetilde{B}_{n,l}(r)]$ for all points
$\widetilde{B}_{6,0}$	{0,2,3}	$1.4 \cdot 10^{-5}$	$1.4 \cdot 10^{-5}$
$\widetilde{B}_{6,2}$	{0,3}	$2.5 \cdot 10^{-5}$	$1.3 \cdot 10^{-4}$
$\widetilde{B}_{7,1}$	{0,2,3}	$7.9 \cdot 10^{-5}$	$2.0 \cdot 10^{-4}$
$\widetilde{B}_{7,3}$	{0,3,5}	$3.4 \cdot 10^{-5}$	$3.4 \cdot 10^{-5}$
$\widetilde{B}_{8,0}$	{0,2,3,4}	$2.2 \cdot 10^{-5}$	$2.2 \cdot 10^{-5}$
$\widetilde{B}_{8,2}$	{0,2,3,4}	$7.4 \cdot 10^{-5}$	$7.4 \cdot 10^{-5}$
$\widetilde{B}_{8,4}$	{0,3,5}	$5.0 \cdot 10^{-5}$	$2.9 \cdot 10^{-4}$
$\widetilde{B}_{9,1}$	{0,2,3,4}	$1.1 \cdot 10^{-4}$	$1.3 \cdot 10^{-4}$
$\widetilde{B}_{9,3}$	{0,2,3,4}	$7.5 \cdot 10^{-5}$	$8.3 \cdot 10^{-3}$
$\widetilde{B}_{9,5}$	{0,3,5}	$1.4 \cdot 10^{-4}$	$1.4 \cdot 10^{-4}$
